# Genetic polymorphisms in DNA repair and damage response genes and late normal tissue complications of radiotherapy for breast cancer

**DOI:** 10.1038/sj.bjc.6605036

**Published:** 2009-04-14

**Authors:** J Chang-Claude, C B Ambrosone, C Lilla, S Kropp, I Helmbold, D von Fournier, W Haase, M-L Sautter-Bihl, F Wenz, P Schmezer, O Popanda

**Affiliations:** 1 Division of Cancer Epidemiology, German Cancer Research Center, Heidelberg, Germany; 2 Department of Cancer Prevention and Control, Roswell Park Cancer Institute, Buffalo, NY, USA; 3 Department of Gynecological Radiology, University Hospital Heidelberg, Heidelberg, Germany; 4 Clinic for Radiotherapy and Radiooncology, St Vincentius-Clinics Karlsruhe, Karlsruhe, Germany; 5 Clinic for Radiotherapy, Municipal Hospital Karlsruhe, Karlsruhe, Germany; 6 Department of Radiation Oncology, University Hospital Mannheim, Mannheim, Germany; 7 Division of Epigenomics and Cancer Risk Factors, German Cancer Research Center, Heidelberg, Germany

**Keywords:** TP53, cosmesis, late side effects, radiotherapy, telangiectasia, fibrosis

## Abstract

Breast-conserving surgery followed by radiotherapy is effective in reducing recurrence; however, telangiectasia and fibrosis can occur as late skin side effects. As radiotherapy acts through producing DNA damage, we investigated whether genetic variation in DNA repair and damage response confers increased susceptibility to develop late normal skin complications. Breast cancer patients who received radiotherapy after breast-conserving surgery were examined for late complications of radiotherapy after a median follow-up time of 51 months. Polymorphisms in genes involved in DNA repair (*APEX1, XRCC1, XRCC2, XRCC3, XPD*) and damage response (*TP53, P21*) were determined. Associations between telangiectasia and genotypes were assessed among 409 patients, using multivariate logistic regression. A total of 131 patients presented with telangiectasia and 28 patients with fibrosis. Patients with variant *TP53* genotypes either for the Arg72Pro or the PIN3 polymorphism were at increased risk of telangiectasia. The odds ratios (OR) were 1.66 (95% confidence interval (CI): 1.02–2.72) for 72Pro carriers and 1.95 (95% CI: 1.13–3.35) for PIN3 A2 allele carriers compared with non-carriers. The *TP53* haplotype containing both variant alleles was associated with almost a two-fold increase in risk (OR 1.97, 95% CI: 1.11–3.52) for telangiectasia. Variants in the *TP53* gene may therefore modify the risk of late skin toxicity after radiotherapy.

Radiotherapy is commonly applied after breast-conserving surgery to reduce the risk of locoregional recurrence of breast cancer and has been shown to be as effective as radical mastectomy (Fisher *et al*, 2002). Although standard radiation therapy is well tolerated by the majority of patients, late normal tissue complications arising from the intrinsic sensitivity of normal tissue, and correlated poor cosmetic results, remain as health concerns of treated breast cancer patients over time (Cetintas *et al*, 2002; Deutsch and Flickinger, 2003; Smith and Ross, 2004). The process of endothelium reconstruction is radiation dose-dependent, progresses over months and years and leads to increases in the severity of both telangiectasia and fibrosis (Bentzen *et al*, 1989; Archambeau *et al*, 1995; Chen *et al*, 2006). Telangiectasias are small dilated blood vessels near the surface of the skin and fibrosis is the development of excess fibrous connective tissue leading to induration. There is, however, considerable inter-individual variability in the development of adverse reactions in normal tissue of irradiated patients. Besides duration, radiation dose and schedule (Turesson *et al*, 1996; Hill *et al*, 2001), patient-related factors, such as age, acute skin reaction and lifestyle factors (Bentzen and Overgaard, 1991; Bentzen *et al*., 1996; Turesson *et al*, 1996; Johansen *et al*, 2002; Deutsch and Flickinger, 2003; Chen *et al*, 2006; Lilla *et al*, 2007), as well as genetic susceptibility (Bentzen and Overgaard, 1994; Chang-Claude *et al*, 2005; Andreassen *et al*, 2006; Popanda *et al*, 2008) have been implicated. Sensitivity to radiation exposure is suggested to be a complex, polygenic trait, which results from the interaction of a number of genes in different cellular pathways (Travis, 2007).

As radiation therapy exerts its cytotoxic effects through damage to cells, proteins and DNA, the individual capacity to repair damaged DNA may modify the response of the normal tissue. Radiation-induced DNA damage is diverse and therefore nearly all DNA repair pathways might be involved in its removal, especially repair of double-strand breaks through mechanisms such as homologous recombination and non-homologous end joining (Jeggo and Lobrich, 2006). In addition, nucleotide and base-excision repair play an important role, mainly in the repair of oxidative DNA damage (Hoeijmakers, 2001).

Furthermore, the complex response to ionising radiation requires the expression and activity of the p53 pathway (Gudkov and Komarova, 2003). The p53 protein is activated through phosphorylation by radiation DNA damage-induced kinases, including ataxia telangiectasia-mutated and the DNA-dependent protein kinase (Banin *et al*, 1998; Fei and El-Deiry, 2003; Schwartz, 2007). Activated p53 protein has various downstream targets, including genes involved in cell-cycle regulation, apoptosis, and DNA repair. Regulation of these processes by p53 controls the cellular response to ionising radiation-induced damage. *p21* is a critical cell-cycle checkpoint gene, regulated tightly by p53. As soon as DNA is damaged by radiation, binding of p53 protein induces transcription of the downstream gene *p21*, which stops cells from entering into the S phase (Robles *et al*, 2002). p21, together with p53, is directly involved in G1/S checkpoint control in response to ionising radiation (Dotto, 2000).

We therefore evaluated the association between several putative functional polymorphisms in six genes involved in DNA repair and two damage response genes and development of late normal tissue complications in a prospective study of breast cancer patients who received radiotherapy after breast-conserving surgery.

## Material and methods

### Patient population and data collection

The methods of this study have been described earlier (Twardella *et al*, 2003; Chang-Claude *et al*, 2005; Lilla *et al*, 2007). Briefly, women diagnosed with breast cancer who received radiotherapy after breast-conserving surgery were enrolled between June 1998 and March 2001 from four radiotherapy units in Germany (Women's Clinic at the University of Heidelberg, St Vincentius Clinic in Karlsruhe, City Hospital in Karlsruhe and University Hospital of Mannheim). Patients who received chemotherapy before or during radiation were not eligible for the study. Information on demographic factors, medical history, and lifestyle factors was obtained through self-administered questionnaires. Details on clinical tumor characteristics and treatment regimen were abstracted from patient records. Informed consent was obtained from all participants, and the study was approved by the ethics committee of the University of Heidelberg, the Institutional Review Board for Roswell Park Cancer Institute, and the US Army Medical Research and Materiel Command Human Subjects Research Review Board.

### Breast irradiation

Details on the radiotherapy regimen (total dose, dose per fraction, treatment time, boost dose) were abstracted from the irradiation protocols. As described earlier (Twardella *et al*, 2003), all patients received a common breast irradiation treatment with conformal tangential irradiation with lateral and medial wedge fields, including CT-based planning, simulation, verification, and quality assurance. At three hospitals, the standard regimen included irradiation of the whole breast, either 50 Gy given in 5 × 2.0 Gy fractions or 50.4 Gy in 5 × 1.8 Gy fractions per week, followed by a photon or electron boost with doses ranging from 5 to 20 Gy. Three patients were treated with brachytherapy (20 or 25 Gy). In the fourth radiation department, patients received 56 Gy of whole breast irradiation in 5 × 2.0 Gy fractions without boost. The biologically effective dose (BED) of radiotherapy relative to an irradiation with a fraction dose of 2.0 Gy, that is the normalised total dose (NTD), was calculated to account for differences in fractionation according to the following formula: 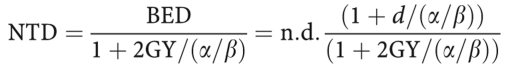 given the number of fractions *n*, the fraction size of *d*, and an *α/β* ratio of 3 Gy for telangiectasia and 2 Gy for fibrosis.

### Follow-up and evaluation of toxicities

The occurrence of acute side effects of radiotherapy was monitored and documented by physicians several times during the study. We have earlier reported on acute radiation-induced toxicity, defined as grade 2c and above (at least one moist desquamation or interruption of radiotherapy due to toxicity), in this patient cohort (Twardella *et al*, 2003; Chang-Claude *et al*, 2005; Ambrosone *et al*, 2006; Popanda *et al*, 2006; Tan *et al*, 2006). Patients were recontacted between June 2003 and July 2005 to assess the occurrence of late adverse effects of radiotherapy and course of disease (relapse, metastases, secondary carcinoma, and death). A self-administered questionnaire similar to that applied at baseline was used to collect information on demographic and epidemiological risk factors, and to record behavior changes that may have occurred after radiotherapy. Patients were examined by the study physician or the treating physician to assess the occurrence of late adverse effects of radiotherapy.

The late side effects were classified according to the RTOG/EORTC late radiation morbidity scoring schema (Seegenschmiedt, 1998) supplemented by LENT-SOMA scores. Patients’ general condition, weight changes, nausea and development of lymphatic edema (arm or breast), and adverse reactions of the skin (telangiectasia), subcutaneous tissue (fibrosis) and other organ tissues (heart, lung, larynx) were recorded. The severity of late effects was scored from 0 to 4, whereby the development of side effects of scores ⩾2 was considered to indicate late normal tissue complications.

### Genotyping assays

Most polymorphisms (see Table 2) were detected by amplification with real-time PCR followed by melting-curve analysis with fluorescence-labeled hybridisation probes in a LightCycler (Roche Diagnostics, Mannheim, Germany) as described earlier (Chang-Claude *et al*, 2005; Popanda *et al*, 2006; Tan *et al*, 2006). The oligonucleotides for analysis of the *XRCC1* -77 polymorphism (rs3213245) were the PCR primers (sense) 5′-ctttagccagcgcaggtcg-3′OH and (antisense) 5′-ccccatgcaggtccctcac-3′OH, sensor 5′-cccgccccctcccactc-3′-FL and anchor 5′-LC Red640-ccctgcccctcggaccccatactc-3′P. The sense primer included a mismatch to avoid stem loops in the amplicon because of the high and repetitive G/C content of the target sequence. PCR primers and probes were designed with the help of Tib Molbiol (Berlin, Germany). Annealing temperature of the primers was 60°C. The PCR was performed for all polymorphisms with Qiagen reagents (Qiagen, Hilden, Germany) in a volume of 10 *μ*l using 10 ng of DNA. Overall, 10% randomly selected samples were analysed by conventional PCR-RFLP to verify the LightCycler results; 100% concordance was found. The insertion of the *TP53* PIN3 polymorphism was identified by standard PCR and electrophoresis (Tan *et al*, 2006). A negative control containing all the reagents but with water instead of the DNA template was included in every amplification set. All genotyping assays were carried out blinded to the clinical diagnosis. For each polymorphism, PCR fragments of the homozygous wild-type allele, the homozygous variant allele, and one heterozygous sample were sequenced.

### Statistical analysis

Significant differences in distribution of genotypes by presence of late skin toxicities (scores ⩾2) were tested by the *χ*^2^ and Fischer's exact tests. Each polymorphism was tested for deviation from Hardy–Weinberg equilibrium by comparing the observed and expected genotype frequencies using the *χ*^2^-test with one degree of freedom. Multivariate unconditional logistic regression analysis was used to assess the association of genotypes with occurrence of late complications of radiotherapy. Odds ratios (OR) and 95% confidence intervals (CI) were computed using the LOGISTIC procedure in SAS version 9.1 (SAS Institute Inc., Cary, NC, USA). Possible effect modification of genotype associations by other covariables was evaluated by the log likelihood ratio test comparing models with and without the first-order interaction terms. All tests were two-sided and considered to be statistically significant with a *P*-value of ⩽0.05. The logistic regression analysis was performed only for the occurrence of telangiectasia, excluding seven patients who developed fibrosis with a score ⩾2 but not telangiectasia. Multivariate models included NTD, age at the time of late toxicity evaluation, and follow-up time since end of radiotherapy. Stepwise backward elimination with *P*⩽0.03 as threshold was used to develop a final model to control for potential confounders. The final model included, in addition hospital facility, acute skin toxicity, a history of hypertension and allergy, skin type (three categories), pack-years of smoking (0, 1–19, ⩾20), and marital status.

Analyses to assess association between haplotypes and risk for telangiectasia were carried out using the function haplo.glm of the R package haplo.stats, which uses a generalised linear model (glm) allowing for an ambiguous linkage phase (Lake *et al*, 2003). The most common haplotype was used as the referent. Possible effect modification of haplotype associations by other covariables were evaluated by the likelihood ratio test. As this was a hypothesis generating study, significance level was defined at *P*<0.05 although 13 SNPs were tested.

## Results

Data on late effects of radiotherapy as well as information on demographic and epidemiological factors were available for 421 breast cancer patients, as reported earlier (Lilla *et al*, 2007; Kuptsova *et al*, 2008). After a median follow-up time of 51 months (range 36–77 months), the most common symptoms of scores ⩾2, which were observed included telangiectasia (32.1%), impairment of the general condition (15.9%), fibrosis (7.1%), lymphatic edema in the arm and breast (6.2%), and pain (5.5%). Of 416 patients (after excluding 3 patients treated with interstitial boost and 2 patients with missing information on fibrosis), 131 patients presented with telangiectasia and 28 with fibrosis of grades ⩾2, whereby 21 patients presented with both adverse reactions. Characteristics of the 409 breast cancer patients who also had genotype data and were included in this analysis (excluding the seven patients presenting with fibrosis only) are shown in Table 1.

We found a significant association between genetic polymorphisms in the *TP53* gene and risk for telangiectasia (Table 2). Compared with non-carriers, patients carrying the variant *TP53* 72Pro allele had an increased risk of adverse effects (OR of 1.66, 95% CI: 1.02–2.72). Carriers of the *TP53* PIN3 A2 allele were also at increased risk of telangiectasia (OR 1.95, 95% CI: 1.13–3.35). None of the other genetic polymorphisms studied showed significant associations with occurrence of telangiectasia.

Strong association (linkage disequilibrium) was found between the *TP53* Arg72Pro and *TP53* PIN3 polymorphisms (*P*<0.001). We therefore investigated haplotype effects of the two *TP53* polymorphisms. Compared with the common ArgA1 haplotype, the ProA2 haplotype containing both variant alleles was associated with a significantly increased OR of 1.97 (95% CI: 1.11–3.52) for telangiectasia (Table 3). Haplotype association analysis for the *XRCC1* and *XPD* genes with data for at least two genetic polymorphisms did not reveal further significant findings.

Further analysis for effect modification yielded differences in the effect of *TP53* on risk for telangiectasia, according to occurrence of acute skin toxicity (moist desquamation). Thirty women (22.9%) had presented with acute skin toxicity during radiotherapy in patients with telangiectasia, and 45 women (16.2%) in those without telangiectasia. The elevated risk of telangiectasia associated with the *TP53* ProA2 haplotype was found only in patients who did not present with acute toxicity during radiotherapy (OR 2.78, 95% CI: 1.44–5.35) and not in those who experienced acute skin toxicity during radiotherapy (*P*_heterogeneity_=0.06) (Table 3).

## Discussion

In this study of breast cancer patients treated with radiotherapy after breast-conserving surgery, we found that variants of *TP53* were associated with an increased risk for developing telangiectasia after radiation therapy. Although both variants, TP53 72Pro and PIN3 A2, were associated with elevated risk, the haplotype results suggested that *cis* effects of the two variants may be most relevant.

Two of the many p53 functions may be important in modulating radiosensitivity. Growth arrest mediated by p53 plays an important role in inhibiting mitotic cell death in epithelia of the small intestine of mice and, thus, is thought to reduce radiation toxicity in these animals (Komarova *et al*, 2004). Also, apoptosis and cell death by mitotic catastrophe have been recognised as an important response to radiation in many cells (Dewey *et al*, 1995; Weber and Wenz, 2002; Komarova *et al*, 2004) as they remove heavily damaged cells from the tissue. Functional analysis of the two *TP53* variants in codon 72 showed that this polymorphism might modulate these two responses. The 72Arg form induced apoptosis more efficiently than the 72Pro form. In contrast, the 72Pro form appeared to induce a higher level of G1 arrest than the 72Arg form giving time to repair (Thomas *et al*, 1999; Dumont *et al*, 2003; Pim and Banks, 2004). Consequently, the 72Pro p53 protein was found to be more efficient in specifically activating p53-dependent DNA repair target genes, and cells carrying the 72Pro allele had significantly higher DNA repair capacity (Siddique and Sabapathy, 2006). Although it is unclear which of the functional differences between the codon 72 polymorphic alleles is more important, our results could be explained by the lower efficiency with which the 72Pro form induced apoptosis of heavily damaged cells after radiation. Repair and reconstitution of the normal tissue function might be incomplete over time in the presence of these cells, leading to late adverse effects which become visible as telangiectasia, a disturbance of the blood vessels.

We also observed an independent effect of the *TP53* PIN3 polymorphism on the risk of late radiation toxicity, but the results of the haplotype analysis suggested the strongest effect on risk conferred by the haplotype containing both variant alleles. The functional significance of *TP53* PIN3 has remained largely unexplored. Our haplotype analysis revealed further that the strongest risk effect of the 72ProA2 haplotype was visible in patients who did not develop severe acute side effects during radiotherapy. We proposed that the Pro allele carriers experienced reduced cell loss by apoptosis and, potentially, mitotic catastrophe during therapy and were, thus, protected from severe acute side effects as we found in our analysis of acute side effects (Tan *et al*, 2006). This protective effect may turn out as a risk factor for late side effects when the irradiated tissue is observed over a longer time. More analyses of the *in vivo* and *in vitro* effects of the *TP53* Arg72Pro and PIN3 polymorphisms are needed, however, before we can apply these *TP53* variants as predictive markers for late side effects of radiotherapy.

p21 plays a direct role in mediating irradiation-induced G1 arrest, with p53 as the transcription factor in this process. This mechanism indicates a possible combined effect of polymorphisms in the two genes. However, p53 may modulate response to radiation damage in the G1 phase of the cell cycle through mechanisms independent of p53-mediated transcriptional activation of p21 and cell-cycle arrest (Mazzatti *et al*, 2005). We did not observe a significant effect of *p21* Ser31Arg polymorphism on the risk of late skin toxicity. Other studies also failed to find an association of this variant with risk or prognosis of breast cancer (Keshava *et al*, 2002; Azzato *et al*, 2008).

In addition, ten polymorphisms causing an amino acid change in six different DNA repair genes were investigated for associations with telangiectasia, but no significant effects were detected. The *XRCC1* Arg399Gln polymorphism has been reported to be associated with telangiectasia but not with fibrosis, particularly in patients who did not receive a boost, albeit based on 167 patients of whom 39 presented with telangiectasia (Giotopoulos *et al*, 2007). This polymorphism was also not found to be associated with severe grade 3 fibrosis after irradiation of the breast (Andreassen *et al*, 2006). A further study, which did not differentiate between early and late adverse reaction to radiotherapy, reported an elevated risk in women carrying both the variant alleles of the Arg194Trp and the Arg399Gln polymorphisms (Moullan *et al*, 2003) and a protective effect for the T-C-G-G haplotype determined by all four *XRCC1* genetic polymorphisms, -77T>C, Arg194Trp, Arg280His, and Arg399Gln (Brem *et al*, 2006). Although the results appear divergent, the studies differ in the specific type(s) of adverse reactions being studied, the length of follow-up for side effects, and adjustment for patient-related factors; therefore, comparison of the findings is problematic. Polymorphisms in *XRCC3* and *APEX1* were studied in breast cancer patients receiving radiotherapy (summarised in Chistiakov *et al*, 2008; Popanda *et al*, 2008). Consistent with our null results, all of these studies failed to show a contribution of these SNPs to the risk of adverse reactions after radiotherapy, implying that they may not be promising candidates for predicting late radiosensitivity.

To our knowledge, this is the first epidemiological study on the two *TP53* genetic variants as predictors of late tissue reactions to radiation therapy. However, both the *TP53* codon 72 and intron 3 variants have been found to be associated with poorer prognosis of non-small cell lung cancer (Boldrini *et al*, 2008). Patients receiving chemoradiotherapy for advanced head and neck cancer were found to have higher response rates and survival when their tumors expressed the proapoptotic 72 Arg allele (Sullivan *et al*, 2004).

This study has a number of strengths. Breast cancer patients from this cohort were treated similarly, with radiation dosage carefully assessed, and patients were followed prospectively. Improved radiation techniques at the time of patient recruitment, as well as retrieval of individual irradiation dose methods and records, allowed for proper calculations of BED. The phenotype was precisely defined using the standardised scoring system for late toxicity. In addition, we accounted for patient- and treatment-related factors that influenced risk for telangiectasia when assessing the effect of the genetic variants.

Both telangiectasia and subcutaneous fibrosis are among the most common long-term skin side effects of radiation therapy. Owing to differences in physiological response to radiation of the various skin layers involved and thereby possible differing genetic susceptibility, we opted to restrict the present analysis to telangiectasia because of the limited occurrence of fibrosis and therefore restricted power. Progressive nature of these complications, together with longer time to follow-up, may permit later analyses of late normal tissue complications in this cohort in the future.

In conclusion, this prospective study showed that variants in the *TP53* gene are associated with risk of late skin toxicity after accounting for patient-related factors and treatment modalities. As this is the first report on the involvement of p53 in late skin adverse effects, replication of these findings in other studies is encouraged. Advances in the search for biomarkers of radiation-induced late skin side effects may lead to improved treatment choices for breast cancer patients, and improve cosmetic outcome as well as quality of life after surviving breast cancer.

## Figures and Tables

**Table 1:** Clinical and demographic characteristics of the breast cancer patients

**Characteristics**	**Mean (s.d.)**	**Range**
Age late toxicities (years)^a^	60.6 (8.57)	27–88
Age radiotherapy (years)^b^	64.7 (8.59)	31–91
Total radiation dose (Gy)^c^	61.8 (4.10)	51–71
Follow-up time (months)	51.4 (6.81)	36–77
		
	**Frequency**	**Percent**
*Body mass index (kg/m* *^2^)*		
<25	182	44.5
25–30	161	39.4
>30	66	16.1
		
*Tumor stage status*		
*In situ*	36	8.8
1	277	67.7
2	92	22.5
Other or unknown	1	0.2
		
*Lymph node status*		
0	314	76.8
1	57	13.9
Unknown	38	9.3
		
*Metastasis status*		
0	261	63.8
1	1	0.2
Unknown	147	35.9
		
*Boost therapy type*		
Photon	275	67.2
Electron	94	23.0
No boost	40	9.8
		
*Radiotherapy clinic*		
University of Heidelberg Women's Clinic	228	55.8
Karlsruhe St Vincentius clinic	96	23.5
Karlsruhe City Hospital	60	14.7
University Hospital of Mannheim	25	6.1

a Age at the time of late toxicities evaluation.

b Age at the end of radiation therapy.

c Includes irradiation to the whole breast and boost application.

**Table 2:** Association between polymorphisms in DNA repair and cell-cycle genes and risk of developing late skin toxicity (telangiectasia) with score ⩾2 after radiotherapy

		**Patients without telangiectasia**		**Patients with telangiectasia**			
**Gene polymorphism**	**Genotype**	***N*=278**	**%**	***N*=131**	**%**	**OR^a^**	**95% CI**
*APEX1*	TT	71	25.9	39	30.7	1.00	
Asp148Gln	TG	134	48.9	65	51.2	1.03	0.58–1.83
*rs3136820*	GG	69	25.2	23	18.1	0.66	0.33–1.32
	TG+GG	203	74.1	88	69.3	0.90	0.53–1.54
							
*XRCC1*	TT	94	34.2	43	33.9	1.00	
−77 T>C	TC	136	49.5	54	42.5	0.97	0.56–1.67
*rs3213245*	CC	45	16.4	30	23.6	1.87	0.94–3.70
	TC+CC	181	65.8	84	66.1	1.17	0.71–1.95
							
*XRCC1*	CC	242	88.3	117	92.1	1.00	
Arg194Trp	CT	30	11	10	7.9	0.58	0.24–1.40
*rs1799782*	TT	2	0.7	0	0	0	0
	CT+TT	32	11.7	10	8	0.57	0.24–1.38
							
*XRCC1*	GG	244	88.4	118	92.9	1.00	
Arg280His	GA	30	10.9	9	7.1	0.49	0.19–1.24
*rs25489*	AA	2	0.7	0	0	0	0
	GA+AA	32	11.6	9	7.1	0.43	0.17–1.09
							
*XRCC1*	GG	112	40.6	50	39.4	1.00	
Arg399Gln	GA	120	43.5	63	49.6	1.09	0.65–1.82
*rs25487*	AA	44	15.9	14	11	0.63	0.29–1.37
	GA+AA	164	59.4	77	60.6	0.96	0.59–1.57
							
*XRCC2*	GG	236	85.5	113	89	1.00	
Arg188His	GA	38	13.8	13	10.2	0.83	0.39–1.76
*rs3218536*	AA	2	0.7	1	0.8	1.05	0.08–13.93
	GA+AA	40	14.5	14	11	0.84	0.41–1.74
							
*XRCC3*	CC	104	38	45	35.4	1.00	
Thr241Met	CT	126	46	63	49.6	1.05	0.62–1.79
*rs861539*	TT	44	16.1	19	15	1.12	0.53–2.40
	CT+TT	170	62	82	64.6	1.07	0.65–1.77
							
*NBS1*	GG	120	43.5	53	41.7	1.00	
Glu185Gln	GC	137	49.6	58	45.7	0.92	0.55–1.54
*rs1805794*	CC	19	6.9	16	12.6	2.14	0.88–5.19
	GC+CC	156	56.5	74	58.3	1.06	0.65–1.72
							
*XPD*	GG	120	43.8	42	33.3	1.00	
Asp312Asn	GA	117	42.7	69	54.8	1.51	0.89–2.55
*rs1799793*	AA	37	13.5	15	11.9	0.91	0.41–2.01
	GA+AA	154	56.2	84	66.6	1.36	0.82–2.24
							
*XPD*	AA	109	39.6	42	33.3	1.00	
Lys751/Gln	AC	133	48.4	65	51.6	1.15	0.68–1.95
*rs13181*	CC	33	12	19	15.1	1.21	0.57–2.58
	AC+CC	166	60.4	84	66.6	1.16	0.70–1.92
							
*P21*	CC	242	87.7	110	86.6	1.00	
Ser31Arg	CA	31	11.2	17	13.4	1.54	0.71–3.32
*rs1801270*	AA	3	1.1	0	0	0	0
	CA+AA	34	12.3	17	13.4	1.27	0.60–2.68
							
*TP53*	GG	160	58.0	64	50.4	1.00	
Arg72Pro	GC	96	34.8	49	38.6	1.67	0.98–2.83
*rs1042522*	CC	20	7.3	14	11	1.62	0.71–3.70
	GC+CC	116	40	63	49.6	1.66	1.02–2.71
							
*TP53*	A1A1	214	77.6	87	68.5	1.00	
PIN3	A1A2	56	20.3	40	31.5	2.14	1.23–3.71
	A2A2	6	2.2	0	0	0	0
	A1A2+A2A2	62	22.4	40	31.5	1.95	1.13–3.37
							

CI=confidence interval; NTD=normalised total dose; OR=odds ratio.

a Adjusted for NTD, age at the time of late toxicities evaluation, time since radiotherapy (months), clinic, acute skin toxicity, high blood pressure, allergy, pack-years (never, <20, ⩾20), skin type (always/moderate/seldom sunburn), clinic, marital status (single/divorced/widowed, married/partner).

**Table 3:** Reconstructed haplotypes and the association with risk of developing late skin toxicity (telangiectasia) with score ⩾2 after radiotherapy

**Gene**	**Haplotype**	**Frequency**	**OR^a^**	**95% CI**
*All patients*				
*TP53*	GA1	0.71	1	
	GA2	0.03	1.02	0.29–3.63
	CA1	0.16	1.20	0.79–1.82
	CA2^b^	0.11	1.97	1.11–3.52
				
*Patients without acute toxicity during radiotherapy* c				
*TP53*	GA1	0.71	1	
	GA2	0.02	0.68	0.13–3.60
	CA1	0.16	1.15	0.71–1.85
	CA2	0.12	2.78	1.44–5.37
				
*Patients with acute toxicity during radiotherapy*				
*TP53*	GA1	0.71	1	
	GA2	0.04	1.10	0.09–13.41
	CA1	0.17	1.47	0.52–4.17
	CA2	0.09	0.52	0.11–2.53
				
*All patients*				
*XRCC1*	CCGG	0.41	1	
	TCGG	0.12	1.15	0.66–2.00
	TCGA	0.36	0.78	0.53–1.15
	TTGG	0.05	0.51	0.21–1.24
	Rare^d^	0.07	0.56	0.26–1.20
				
*XPD*	GA	0.56	1	
	GC	0.09	1.25	0.64–2.46
	AA	0.06	1.14	0.55–2.38
	AC	0.29	1.09	0.74–1.62

CI=confidence interval; NTD=normalised total dose; OR=odds ratio.

a Adjusted for NTD, age the time of late toxicities evaluation, time since radiotherapy (months), clinic, acute skin toxicity, high blood pressure, allergy, pack-years (never, <20, ⩾20), skin type (always/moderate/seldom sunburn), clinic, marital status (single/divorced/widowed, married/partner).

b A2 allele carries a duplication of 16 bp in intron 3.

c *P*=0.06 for effect heterogeneity according to occurrence of acute skin toxicity.

d Composed of haplotypes with frequencies below 5%.

## References

[bib1] Ambrosone CB, Tian C, Ahn J, Kropp S, Helmbold I, von FD, Haase W, Sautter-Bihl ML, Wenz F, Chang-Claude J (2006) Genetic predictors of acute toxicities related to radiation therapy following lumpectomy for breast cancer: a case-series study. Breast Cancer Res 8: R40 16848913 10.1186/bcr1526 PMC1779469

[bib2] Andreassen CN, Overgaard J, Alsner J, Overgaard M, Herskind C, Cesaretti JA, Atencio DP, Green S, Formenti SC, Stock RG, Rosenstein BS (2006) ATM sequence variants and risk of radiation-induced subcutaneous fibrosis after postmastectomy radiotherapy. Int J Radiat Oncol Biol Phys 64: 776–783 16338099 10.1016/j.ijrobp.2005.09.014

[bib3] Archambeau JO, Pezner R, Wasserman T (1995) Pathophysiology of irradiated skin and breast. Int J Radiat Oncol Biol Phys 31: 1171–1185 7713781 10.1016/0360-3016(94)00423-I

[bib4] Azzato EM, Driver KE, Lesueur F, Shah M, Greenberg D, Easton DF, Teschendorff AE, Caldas C, Caporaso NE, Pharoah PD (2008) Effects of common germline genetic variation in cell cycle control genes on breast cancer survival: results from a population-based cohort. Breast Cancer Res 10: R47 18507837 10.1186/bcr2100 PMC2481496

[bib5] Banin S, Moyal L, Shieh S, Taya Y, Anderson CW, Chessa L, Smorodinsky NI, Prives C, Reiss Y, Shiloh Y, Ziv Y (1998) Enhanced phosphorylation of p53 by ATM in response to DNA damage. Science 281: 1674–1677 9733514 10.1126/science.281.5383.1674

[bib6] Bentzen SM, Overgaard J (1994) Patient-to-Patient Variability in the Expression of Radiation-Induced Normal Tissue Injury. Semin Radiat Oncol 4: 68–80 10717093 10.1053/SRAO00400068

[bib7] Bentzen SM, Overgaard M (1991) Relationship between early and late normal-tissue injury after postmastectomy radiotherapy. Radiother Oncol 20: 159–165 1852907 10.1016/0167-8140(91)90092-u

[bib8] Bentzen SM, Skoczylas JZ, Overgaard M, Overgaard J (1996) Radiotherapy-related lung fibrosis enhanced by tamoxifen. J Natl Cancer Inst 88: 918–922 8656444 10.1093/jnci/88.13.918

[bib9] Bentzen SM, Thames HD, Overgaard M (1989) Latent-time estimation for late cutaneous and subcutaneous radiation reactions in a single-follow-up clinical study. Radiother Oncol 15: 267–274 2772254 10.1016/0167-8140(89)90095-9

[bib10] Boldrini L, Gisfredi S, Ursino S, Lucchi M, Greco G, Mussi A, Donati V, Fontanini G (2008) Effect of the p53 codon 72 and intron 3 polymorphisms on non-small cell lung cancer (NSCLC) prognosis. Cancer Invest 26: 168–172 18259947 10.1080/07357900701788023

[bib11] Brem R, Cox DG, Chapot B, Moullan N, Romestaing P, Gerard JP, Pisani P, Hall J (2006) The XRCC1 -77T->C variant: haplotypes, breast cancer risk, response to radiotherapy and the cellular response to DNA damage. Carcinogenesis 27: 2469–2474 16829685 10.1093/carcin/bgl114

[bib12] Cetintas SK, Ozkan L, Kurt M, Saran A, Tasdelen I, Tolunay S, Topal U, Engin K (2002) Factors influencing cosmetic results after breast conserving management (Turkish experience). Breast 11: 72–80 14965649 10.1054/brst.2001.0372

[bib13] Chang-Claude J, Popanda O, Tan XL, Kropp S, Helmbold I, von Fournier D, Haase W, Sautter-Bihl ML, Wenz F, Schmezer P, Ambrosone CB (2005) Association between polymorphisms in the DNA repair genes, XRCC1, APE1, and XPD and acute side effects of radiotherapy in breast cancer patients. Clin Cancer Res 11: 4802–4809 16000577 10.1158/1078-0432.CCR-04-2657

[bib14] Chen PY, Vicini FA, Benitez P, Kestin LL, Wallace M, Mitchell C, Pettinga J, Martinez AA (2006) Long-term cosmetic results and toxicity after accelerated partial-breast irradiation: a method of radiation delivery by interstitial brachytherapy for the treatment of early-stage breast carcinoma. Cancer 106: 991–999 16421922 10.1002/cncr.21681

[bib15] Chistiakov DA, Voronova NV, Chistiakov PA (2008) Genetic variations in DNA repair genes, radiosensitivity to cancer and susceptibility to acute tissue reactions in radiotherapy-treated cancer patients. Acta Oncol 47: 809–824 18568480 10.1080/02841860801885969

[bib16] Deutsch M, Flickinger JC (2003) Patient characteristics and treatment factors affecting cosmesis following lumpectomy and breast irradiation. Am J Clin Oncol 26: 350–353 12902883 10.1097/01.COC.0000020589.75948.E7

[bib17] Dewey WC, Ling CC, Meyn RE (1995) Radiation-induced apoptosis: relevance to radiotherapy. Int J Radiat Oncol Biol Phys 33: 781–796 7591884 10.1016/0360-3016(95)00214-8

[bib18] Dotto GP (2000) p21(WAF1/Cip1): more than a break to the cell cycle? Biochim Biophys Acta 1471: M43–M56 10967424 10.1016/s0304-419x(00)00019-6

[bib19] Dumont P, Leu JI, Della III PA, George DL, Murphy M (2003) The codon 72 polymorphic variants of p53 have markedly different apoptotic potential. Nat Genet 33: 357–365 12567188 10.1038/ng1093

[bib20] Fei P, El-Deiry WS (2003) P53 and radiation responses. Oncogene 22: 5774–5783 12947385 10.1038/sj.onc.1206677

[bib21] Fisher B, Anderson S, Bryant J, Margolese RG, Deutsch M, Fisher ER, Jeong JH, Wolmark N (2002) Twenty-year follow-up of a randomized trial comparing total mastectomy, lumpectomy, and lumpectomy plus irradiation for the treatment of invasive breast cancer. N Engl J Med 347: 1233–1241 12393820 10.1056/NEJMoa022152

[bib22] Giotopoulos G, Symonds RP, Foweraker K, Griffin M, Peat I, Osman A, Plumb M (2007) The late radiotherapy normal tissue injury phenotypes of telangiectasia, fibrosis and atrophy in breast cancer patients have distinct genotype-dependent causes. Br J Cancer 96: 1001–1007 17325707 10.1038/sj.bjc.6603637 PMC2360097

[bib23] Gudkov AV, Komarova EA (2003) The role of p53 in determining sensitivity to radiotherapy. Nat Rev Cancer 3: 117–129 12563311 10.1038/nrc992

[bib24] Hill RP, Rodemann HP, Hendry JH, Roberts SA, Anscher MS (2001) Normal tissue radiobiology: from the laboratory to the clinic. Int J Radiat Oncol Biol Phys 49: 353–365 11173128 10.1016/s0360-3016(00)01484-x

[bib25] Hoeijmakers JH (2001) Genome maintenance mechanisms for preventing cancer. Nature 411: 366–374 11357144 10.1038/35077232

[bib26] Jeggo P, Lobrich M (2006) Radiation-induced DNA damage responses. Radiat Prot Dosimetry 122: 124–127 17351270 10.1093/rpd/ncl495

[bib27] Johansen J, Overgaard J, Rose C, Engelholm SA, Gadeberg CC, Kjaer M, Kamby C, Juul-Christensen J, Blichert-Toft M, Overgaard M (2002) Cosmetic outcome and breast morbidity in breast-conserving treatment--results from the Danish DBCG-82TM national randomized trial in breast cancer. Acta Oncol 41: 369–380 12234030 10.1080/028418602760169433

[bib28] Keshava C, Frye BL, Wolff MS, McCanlies EC, Weston A (2002) Waf-1 (p21) and p53 polymorphisms in breast cancer. Cancer Epidemiol Biomarkers Prev 11: 127–130 11815410

[bib29] Komarova EA, Kondratov RV, Wang K, Christov K, Golovkina TV, Goldblum JR, Gudkov AV (2004) Dual effect of p53 on radiation sensitivity *in vivo*: p53 promotes hematopoietic injury, but protects from gastro-intestinal syndrome in mice. Oncogene 23: 3265–3271 15064735 10.1038/sj.onc.1207494

[bib30] Kuptsova N, Chang-Claude J, Kropp S, Helmbold I, Schmezer P, von FD, Haase W, Sautter-Bihl ML, Wenz F, Onel K, Ambrosone CB (2008) Genetic predictors of long-term toxicities after radiation therapy for breast cancer. Int J Cancer 122: 1333–1339 18027873 10.1002/ijc.23138

[bib31] Lake SL, Lyon H, Tantisira K, Silverman EK, Weiss ST, Laird NM, Schaid DJ (2003) Estimation and tests of haplotype-environment interaction when linkage phase is ambiguous. Hum Hered 55: 56–65 12890927 10.1159/000071811

[bib32] Lilla C, Ambrosone CB, Kropp S, Helmbold I, Schmezer P, von FD, Haase W, Sautter-Bihl ML, Wenz F, Chang-Claude J (2007) Predictive factors for late normal tissue complications following radiotherapy for breast cancer. Breast Cancer Res Treat 106: 143–150 17221151 10.1007/s10549-006-9480-9

[bib33] Mazzatti DJ, Lee YJ, Helt CE, O′Reilly MA, Keng PC (2005) p53 modulates radiation sensitivity independent of p21 transcriptional activation. Am J Clin Oncol 28: 43–50 15685034 10.1097/01.coc.0000139484.51715.5a

[bib34] Moullan N, Cox DG, Angele S, Romestaing P, Gerard JP, Hall J (2003) Polymorphisms in the DNA repair gene XRCC1, breast cancer risk, and response to radiotherapy. Cancer Epidemiol Biomarkers Prev 12: 1168–1174 14652276

[bib35] Pim D, Banks L (2004) p53 polymorphic variants at codon 72 exert different effects on cell cycle progression. Int J Cancer 108: 196–199 14639602 10.1002/ijc.11548

[bib36] Popanda O, Marquardt JU, Chang-Claude J, Schmezer P (2008) Genetic variation in normal tissue toxicity induced by ionizing radiation. Mut Res; e-pub ahead of print 5 November 2008, PMID: 19022265; doi: 10.1016/j.mrfmmm.2008.10.014 10.1016/j.mrfmmm.2008.10.014 19022265

[bib37] Popanda O, Tan XL, Ambrosone CB, Kropp S, Helmbold I, von Fournier D, Haase W, Sautter-Bihl ML, Wenz F, Schmezer P, Chang-Claude J (2006) Genetic polymorphisms in the DNA double-strand break repair genes XRCC3, XRCC2, and NBS1 are not associated with acute side effects of radiotherapy in breast cancer patients. Cancer Epidemiol Biomarkers Prev 15: 1048–1050 16702393 10.1158/1055-9965.EPI-06-0046

[bib38] Robles AI, Linke SP, Harris CC (2002) The p53 network in lung carcinogenesis. Oncogene 21: 6898–6907 12362272 10.1038/sj.onc.1205563

[bib39] Schwartz JL (2007) Variability: the common factor linking low dose-induced genomic instability, adaptation and bystander effects. Mutat Res 616: 196–200 17145066 10.1016/j.mrfmmm.2006.11.016

[bib40] Seegenschmiedt MH (1998) Interdisciplinary documentation of treatment side effects in oncology. Present status and perspectives. Strahlenther Onkol 174(Suppl 3): 25–29 9830452

[bib41] Siddique M, Sabapathy K (2006) Trp53-dependent DNA-repair is affected by the codon 72 polymorphism. Oncogene 25: 3489–3500 16462765 10.1038/sj.onc.1209405

[bib42] Smith IE, Ross GM (2004) Breast radiotherapy after lumpectomy--no longer always necessary. N Engl J Med 351: 1021–1023 15342811 10.1056/NEJMe048173

[bib43] Sullivan A, Syed N, Gasco M, Bergamaschi D, Trigiante G, Attard M, Hiller L, Farrell PJ, Smith P, Lu X, Crook T (2004) Polymorphism in wild-type p53 modulates response to chemotherapy *in vitro* and *in vivo*. Oncogene 23: 3328–3337 15077186 10.1038/sj.onc.1207428

[bib44] Tan XL, Popanda O, Ambrosone CB, Kropp S, Helmbold I, von FD, Haase W, Sautter-Bihl ML, Wenz F, Schmezer P, Chang-Claude J (2006) Association between TP53 and p21 genetic polymorphisms and acute side effects of radiotherapy in breast cancer patients. Breast Cancer Res Treat 97: 255–262 16331344 10.1007/s10549-005-9119-2

[bib45] Thomas M, Kalita A, Labrecque S, Pim D, Banks L, Matlashewski G (1999) Two polymorphic variants of wild-type p53 differ biochemically and biologically. Mol Cell Biol 19: 1092–1100 9891044 10.1128/mcb.19.2.1092 PMC116039

[bib46] Travis EL (2007) Genetic susceptibility to late normal tissue injury. Semin Radiat Oncol 17: 149–155 17395045 10.1016/j.semradonc.2006.11.011

[bib47] Turesson I, Nyman J, Holmberg E, Oden A (1996) Prognostic factors for acute and late skin reactions in radiotherapy patients. Int J Radiat Oncol Biol Phys 36: 1065–1075 8985028 10.1016/s0360-3016(96)00426-9

[bib48] Twardella D, Popanda O, Helmbold I, Ebbeler R, Benner A, von Fournier D, Haase W, Sautter-Bihl ML, Wenz F, Schmezer P, Chang-Claude J (2003) Personal characteristics, therapy modalities and individual DNA repair capacity as predictive factors of acute skin toxicity in an unselected cohort of breast cancer patients receiving radiotherapy. Radiother Oncol 69: 145–153 14643951 10.1016/s0167-8140(03)00166-x

[bib49] Weber KJ, Wenz F (2002) p53, apoptosis and radiosensitivity—experimental and clinical data. Onkologie 25: 136–141 12006764 10.1159/000055223

